# *PCDH19* in Males: Are Hemizygous Variants Linked to Autism?

**DOI:** 10.3390/genes14030598

**Published:** 2023-02-27

**Authors:** Eliane Chouery, Jana Makhlouf, Wassim Daoud Khatoun, Cybel Mehawej, Andre Megarbane

**Affiliations:** 1 Department of Human Genetics, Gilbert and Rose-Marie Chagoury School of Medicine, Lebanese American University, Byblos 1102-2801, Lebanon; 2 Gilbert and Rose-Marie Chagoury School of Medicine, Lebanese American University, Byblos 1102-2801, Lebanon; 3 Institut Jérôme Lejeune, 75015 Paris, France

**Keywords:** *PCDH19*, autism spectrum disorders, whole-exome sequencing, variant of unknown significance

## Abstract

Background: Autism spectrum disorder (ASD) is a complex developmental disability that impairs the social communication and interaction of affected individuals and leads to restricted or repetitive behaviors or interests. ASD is genetically heterogeneous, with inheritable and de novo genetic variants in more than hundreds of genes contributing to the disease. However, these account for only around 20% of cases, while the molecular basis of the majority of cases remains unelucidated as of yet. Material and methods: Two unrelated Lebanese patients, a 7-year-old boy (patient A) and a 4-year-old boy (patient B), presenting with ASD were included in this study. Whole-exome sequencing (WES) was carried out for these patients to identify the molecular cause of their diseases. Results: WES analysis revealed hemizygous variants in *PCDH19* (NM_001184880.1) as being the candidate causative variants: p.Arg787Leu was detected in patient A and p.Asp1024Asn in patient B. PCDH19, located on chromosome X, encodes a membrane glycoprotein belonging to the protocadherin family. Heterozygous *PCDH19* variants have been linked to epilepsy in females with mental retardation (EFMR), while mosaic *PCDH19* mutations in males are responsible for treatment-resistant epilepsy presenting similarly to EFMR, with some reported cases of comorbid intellectual disability and autism. Interestingly, a hemizygous *PCDH19* variant affecting the same amino acid that is altered in patient A was previously reported in a male patient with ASD. Conclusion: Here, we report hemizygous *PCDH19* variants in two males with autism without epilepsy. Reporting further *PCDH19* variants in male patients with ASD is important to assess the possible involvement of this gene in autism.

## 1. Introduction

Autism spectrum disorders (ASD) are a wide constellation of neurodegenerative diseases that present from birth as a deficit in communication, especially in social settings, as well as defects in sensory processing. According to the Diagnostic and Statistical Manual of Mental Disorders, Fifth Edition (DSM-5), ASD is diagnosed in the early developmental period in the presence of persistent social deficits in multiple contexts constituting communication and interaction and repetitive behavioral patterns that are not better explained by other developmental or intellectual disorders [1]. Despite the clear criteria that were detailed in the DSM-5 to diagnose ASD, the clinical presentation of autism remains variable, making the diagnosis of these conditions challenging and delaying it to an advanced age in some cases [2]. A meta-analysis, which was published in 2020 and covered studies from 35 different countries, indicated that the mean age at the time of diagnosis of ASD falls anywhere between 2 years and a half and 20 years of age, with a mean age of 5 years old [3]. Nevertheless, the recommendation set forward by the American Academy of Pediatrics (AAP) suggests screening services for symptoms of ASD be provided to all children at the ages of 18 and 24 months due to the possibility of early detection and the subsequent behavioral, developmental, educational, and psychological interventions that were proven to be impactful [2,4,5]. Regarding its epidemiology, there is a wide variability in the estimates of ASD prevalence due to methodological differences in its determination, rendering its prevalence range between 0.19 in 1000 and 11.6 in 1000 [6]. 

ASD are diagnosed more commonly in males regardless of age stratification, with a ratio of 3 males being diagnosed for every 1 female [7,8]. However, this ratio is decreased in individuals who are known to have an intellectual disability, becoming closer to 2 males for every 1 female [9,10]. However, the reason for this discrepancy remains unclear, with several theories emerging to explain it. Some argue that it is due to an intrinsic protective effect of the female brain against developing autism [11]. On the other hand, some postulate that this discrepancy is simply a result of the underdiagnosis of females due to the variable expression of autistic symptoms in female patients [9].

ASD have been under the spotlight for the last 50 years, with increasing research focusing on the molecular basis implicated in their development. Many environmental factors have been linked to the development of ASD, including but not limited to advanced parental age, maternal physical and mental health, prenatal medication use, and familial socioeconomic factors. Multiple studies have revealed the interaction between those environmental factors and genetic determinants, thus adding to the complexity of ASD [12,13,14,15]. 

Polygenic inheritance appears to be at the core of the disorder’s etiology. Genes that are known to increase the likelihood of ASD occurrence include ones that encode cell-adhesion proteins, synaptic vesicle cycling proteins, and ion transport proteins, with others functioning in mechanisms of epigenetic modulation. To date, most of the data related to susceptibility to ASD were generated from genome-wide association studies (GWAS) that explored common genetic variants, including single-nucleotide polymorphisms (SNPs) or point variants and rare copy number variants (CNVs), which are large genomic deletions and duplications involving multiple genes [16]. In addition, advances in the molecular biology field have enabled the use of next-generation sequencing techniques to detect rare SNPs as well as structural variants that can be directly linked to high-risk autism. Indeed, pathogenic mutations have been reported in elements that regulate these ASD risk genes. Genetic modifications implicated in ASD seem to be heterogeneous and include somatic mosaicism variations and copy number variants. Interestingly, epigenetics also appears to play a role in the pathophysiology of ASD. For example, the chromatin modifier, methyl-CpG binding protein 2 (MeCP2), which is famously known for its association with Rett syndrome, which typically falls into the spectrum of autism, normally regulates genes involved in synaptic function, including protocadherin β 1 (*PCDHB1*) and protocadherin 7 (*PCDH7*) [13,17]. The gene encoding MeCP2 has been found to be hypermethylated at its promoter region in the frontal cortex of ASD patients, thus resulting in the downregulation of its expression [13]. 

Of particular interest, genes encoding clustered protocadherins have been reported in various studies to be implicated as ASD risk genes, with changes including SNPs, CNVs, and different methylation patterns [18]. To our knowledge, although some non-clustered protocadherins, such as protocadherins 8 and 9, have been linked to autism [19], only one report of *PCDH19* mutations has been directly correlated to autism in a male patient [20].

In this case study, we describe two *PCDH19* variants (NM_001184880.1: p.Arg787Leu and p.Asp1024Asn) in two unrelated male patients clinically diagnosed with autism. 

## 2. Material and Methods

### 2.1. Patient

We herein describe two young Lebanese boys referred for clinical investigation of ASD. The DSM-5 criteria were used by pediatric neurologists to clinically assess ASD in the patients. Written informed consent forms were obtained from legally authorized representatives of the patients (parental consent) to participate in this study, and approval was granted for the publication of the findings. 

### 2.2. Isolation of Genomic DNA

EDTA blood samples from all members of the family were collected for genetic studies. DNA was extracted from leucocytes by the standard salt-precipitation methods reported earlier [21].

### 2.3. Whole-Exome Sequencing (WES)

Whole-exome sequencing (WES) was carried out on the patients using standard techniques [22]. Briefly, the exome was captured and enriched using the solution Agilent SureSelect Human All Exon kit version 5.0, and samples were then multiplexed and subjected to sequencing on an Illumina HiSeq 2500 PE100-125. Read files (FASTQ) were generated from the sequencing platform via the manufacturer’s proprietary software. Reads were aligned to the hg19/b37 reference genome using the Burrows–Wheeler Aligner (BWA) package version 0.7.11 [23]. Variant calling was subsequently performed using the Genome Analysis Tool Kit (GATK) version 3.3 [24]. Variants were called using high stringency settings and annotated with VarAFT software 1.61 [25] containing information from dbSNP147 and the Genome Aggregation Database (gnomAD, http://gnomad.broadinstitute.org accessed on 1 August 2022). Only nonsynonymous coding and splicing variants found in the patient were considered. Variant filtering was then performed based on the frequency of the variant in the gnomAD database (<0.01% and <50 heterozygous carriers or <5 homo-/hemizygous carriers) and in our in-house database (<1 homozygous occurrence), whereby only rare variants were selected. The last step in our filtering strategy consisted of excluding all variants with a sequencing depth lower than 15x, as well as those predicted to be benign or likely benign.

### 2.4. Sanger Sequencing

The genomic sequence of *PCDH19* (NM_001184880.1) was obtained from UCSC Genomic Browser. Primers used for PCR amplification were designed using Primer3 software (http://frodo.wi.mit.edu accessed on 30 November 2022) to amplify exon 6 of the PCDH19 gene, including the p.Asp1024Asn detected by WES in the patient B. PCR reactions were performed using Taq DNA polymerase (Invitrogen Life Technologies, Carlsbad, CA, USA). PCR fragments were run on 1% agarose gel. The fragments were purified using a Sigma-Aldrich kit and then sequenced using the Big Dye_Terminator v1.1 Cycle Sequencing Kit (Applied Biosystems, Foster City, CA, USA). The sequence reaction was purified on a Sephadex G50 (Amersham Pharmacia Biotech, Foster City, CA, USA) and then loaded into an ABI3500 system after the addition of Hidi formamide. Electropherograms were analyzed using Sequence Analysis Software version 5.2 (Applied Biosystems, Foster City, CA, USA) and then aligned with the reference sequences using ChromasPro v1.7.6.1 (Technelysium, Queensland, Australia).

## 3. Results

### 3.1. Clinical Presentations

Two unrelated Lebanese patients are included in this study. The first patient is a 7-year-old boy (patient A) born to a 31-year-old mother and a 33-year-old father, while the second patient is a 4-year-old boy (patient B) born to a 30-year-old mother and a 35-year-old father. Both patient A and patient B were born at term after a smooth and uncomplicated pregnancy, labor and delivery to non-consanguineous families. In both families, the mothers were healthy (without any history of diabetes or obesity) and were not exposed during their pregnancies to pesticides or medications. While patient A was the only child in his family, patient B is the second child, with an older unaffected 7-year-old brother. Physical examination at birth demonstrated head circumference, length, and weight that fall within normal ranges. Both patients A and B had no growth delays noted.

Patient A started walking at the age of 12 months. His developmental milestones were normal until the age of 1.5 years, when his parents started noticing some warning signs of autism: he was not responding to them, was avoiding eye contact and was not smiling when they smiled at him. Regarding other developmental milestones, he acquired sphincter control at the age of 3 years. He began to speak a few words at 7 years old. On physical examination, he had no dysmorphic features. However, hyperlaxity of the joints and flat feet were noted.

Patient B started walking at 12 months of age. At the age of 2 years, his parents started noticing some autistic behaviors, mainly the absence of communication and interaction with his environment. Currently, the patient is 4 years old, and he still does not speak, only responds randomly to his name, and avoids eye contact. He has not acquired sphincter control yet. Unlike patient A, he does not present with any hyperlaxity of the joints or dysmorphic features.

In both patients, ophthalmologic and auditory evaluations were normal. Routine laboratory workup, heavy metal blood test screening and metabolic workup testing (blood gases, liver function test, serum lactate, serum ammonia, plasma amino acids, acylcarnitines, urine organic acids, and biotinidase) were found to be within normal limits. Brain magnetic resonance imaging (MRI), as well as electroencephalogram tests (EEG), did not show any abnormalities.

Both patients met the diagnostic criteria for ASD according to the DSM-5, as they have deficits in each of the three areas of social communication and interaction in addition to at least two types of restricted and repetitive behaviors.

### 3.2. Genetic Studies

WES was performed in both patients and led to the identification of 97,838 and 100,485 variants in patients A and B, respectively. Of these, all non-genic, non-splice site, and intronic variants were excluded. The remaining variants were then filtered based on their frequency in databases; only rare variants (present in less than 1% in public databases or in our in-house exome database) were retained. The last step in our filtering strategy consisted of excluding all variants with a sequencing depth lower than 15x, as well as those predicted to be benign or likely benign. This led to the selection of 544 and 610 variants in patients A and B, respectively. These were thoroughly studied in order to select the candidate variants that can be involved in the clinical picture of the patients. Only one gene, the *PCDH19* gene (NM_001184880.1), was selected as a candidate in both patients, whereby each of the included cases carried a hemizygous variant in *PCDH19*, as follows: p.Arg787Leu (c.2360G>T) in Patient A and p.Asp1024Asn (c.3070G>A) in Patient B. Both identified variants are absent in our local database but present a heterozygous state in 2 females out of 181,469 individuals in gnomAD (0.001102%). Segregation analysis performed by Sanger sequencing in family B showed that the X-linked variant (p.Asp1024Asn) was inherited from the carrier mother but was absent in the unaffected brother. Unfortunately, sequencing the variant in family A was not pursued due to the inaccessibility of samples from the mother. The variant p.Arg787Leu, detected in patient A, is located in exon 2 of the *PCDH19* gene and is predicted to be pathogenic based on 10 pathogenic predictions from BayesDel_addAF, BayesDel_noAF, DANN, FATHMM-MKL, LIST-S2, LRT, M-CAP, MVP, Mutation Taster and PrimateAI vs. 3 benign predictions from DEOGEN2, Mutation Assessor and SIFT. Therefore, the p.Arg787Leu variant is classified as a VUS (PM2, PP2, PP3) based on the American College of Medical Genetics (ACMG) classification [20]. Interestingly, a hemizygous *PCDH19* variant affecting the same amino acid (p.Arg787Cys) was previously reported by van Harssel et al., 2013, in a male patient with ASD [20].

The variant p.Asp1024Asn, detected in patient B, is located in exon 6 of the *PCDH19* gene and is predicted to be a VUS based on 9 benign predictions from BayesDel_addAF, DANN, DEOGEN2, LIST-S2, M-CAP, MVP, MutationAssessor, PrimateAI and SIFT vs. 3 pathogenic predictions from FATHMM-MKL, LRT and Mutation Taster. It is also classified as a VUS (PM2, PP2, BP4) based on the ACMG classification [26].

In order to avoid missing any mutation in other candidate genes, WES and coverage data were then checked for a panel of 735 genes implicated in autism and ASD (Supplementary Table S1). This analysis showed that all genes were 100% covered (at a minimum of 20x) and excluded the presence of additional pathogenic point variations in genes known to be involved in similar diseases.

## 4. Discussion

We report herein two different *PCDH19* variants in two unrelated male patients clinically diagnosed with ASD.

*PCDH19*, located on chromosome X, encodes a membrane glycoprotein belonging to the protocadherin family that is the largest subgroup in the cadherin superfamily, particularly in its non-clustered δ2-protocadherin subgroup. This glycoprotein mediates strengthening cell-cell aggregation through homophilic binding in a calcium-dependent manner that is similar to but weaker than that observed with cadherin-dependent adhesion [19,27,28,29]. Although the exact function of *PCDH19* has not been completely established, growing evidence shows its involvement in neurodevelopment through its role in cell migration, axonal outgrowth and synaptogenesis [27,28,30]. This substantial role of protocadherin-19 has been shown to be implicated in early brain morphogenesis through its expression in the anterior neural plate during early neurulation in zebrafish embryo models. Accordingly, the defective brain morphology observed in zebrafish embryos with a defect in *PCDH19* appears to be partially mediated by an impaired cell movement in this plate [28,30]. Early evidence suggests that protocadherin-19 cooperates with N-cadherin to coordinate cell adhesion, and an impairment in either protein results in defective cell motility, with neighboring cells exhibiting a jeopardized coordination of movement. Subsequently, this impaired cellular interaction could indicate a disrupted cellular response to spatial signals, hence resulting in defective cell migration in the anterior neural plate [28]. 

Clinically, genetic variants in *PCDH19* have been linked to a rare newly individualized syndrome: epilepsy in females with mental retardation (EFMR), also known as early infantile epileptic encephalopathy (EIEE9) or *PCDH-19* clustering epilepsy (OMIM #300088), and in fact comes second in the list of genes associated with this disease [31,32]. This sex-limited disease is characterized by the early onset of seizures, usually with varying degrees of intellectual disability [33] and other neurobehavioral features [34]. The spectrum of this intellectual disability ranges from normal cognitive function to severe intellectual impairment, with possible neuropsychiatric presentations including autistic behavior as the most prevalent comorbidity, followed by or accompanied by hyperactivity and/or attention deficit [35]. For years, researchers have been keen to investigate the molecular mechanism behind the unconventional mode of inheritance of X-linked mutations in *PCDH19*, as the disease-specific phenotype predominantly appears in heterozygous females or male mosaic carriers but not in hemizygous males [31,36,37]. Researchers attempting to explain this pattern of inheritance have postulated that the random chromosome X inactivation gives rise to two populations of neurons in a carrier female: one carrying the normal variant of the *PCDH19* gene and the other carrying a mutated variant. The abnormal interaction between these two neural populations, termed “cellular interference”, may be the basis of the pathogenesis of this disease [33]. This role of cellular interference in the pathogenesis of *PCDH19*-associated epilepsy is further supported by a case report describing a male with Klinefelter syndrome (XXY) who is heterozygous for *PCDH19* mutation presenting with early onset epileptic seizures [38]. The mosaic expression of protocadherin-19 could impair functional processes in the brain, knowledge on which remains scarce, and hence explain the aforementioned unique mode of inheritance [36,37,39]. Along these lines, Pederick et al. (2017) reported that, in *PCDH19* girls with clustering epilepsy, differential adhesion affinities result from this differential expression of protocadherin-19 in the developing cortex, thus interfering with the normal arrangement of neuroprogenitor cells [39]. As such, the absence of a disease-specific phenotype in strictly hemizygous males, where *PCDH19* is uniformly removed or aberrantly expressed across cells, could be the consequence of the uniform cell sorting in the developing cortex.

While epilepsy in EFMR patients has been shown to follow this peculiar genotype-phenotype expression pattern, it remains unclear whether it applies to other symptoms of *PCDH19* mutation. Autism spectrum disorder (ASD) has been reported in females with EFMR [34]. In knockout studies on mice, *PCDH19* heterozygous females exhibited decreased sociability, thus indicating autism-like features. Hemizygous males with no expression of *PCDH19* were also demonstrated to have autistic features. This is inconsistent with the expression of epilepsy, which is absent in hemizygous males, and suggests that the pathogenesis of ASD is not related to cellular interference [33]. Therefore, autism could be the only presenting feature of *PCDH19* mutations in hemizygous males.

As stated before, in females, heterozygous X-linked mutations in *PCDH19* have been linked to EFMR, while in males, mosaic *PCDH19* mutations are responsible for treatment-resistant epilepsy presenting similarly to EFMR, with some reported cases of comorbid intellectual disability, including autism and other behavioral problems [40,41]. On the other hand, males with hemizygous *PCDH19* mutations appear to only have a rigid personality. Here we report hemizygous *PCDH19* mutations in two males with autism without epilepsy. Interestingly, one male patient clinically diagnosed with ASD and carrying the *PCDH19* variant p.Arg787Cys affecting the same amino acid that is altered in patient A was reported by van Harssel et al. in 2013 [20]. Furthermore, knockout studies on mice show that *PCDH19* hemizygous males with no expression of *PCDH19* have autistic features, while *PCDH19* heterozygous females exhibit EFMR, including decreased sociability, thus indicating autism-like features as well [33].

In contrast to epilepsy, which was shown to require the interaction between two neuronal populations, one of which carries the *PCDH19* mutation [33], the pathogenesis of autism does not seem to be related to cellular interference. Therefore, autism could be the only presenting feature of *PCDH19* mutations in hemizygous males in the absence of tissue mosaicism. 

In spite of the scarcity of reports on the association between *PCDH19* and autism, the link between these two comes as no surprise as prior genetic analyses of patients with neurodevelopmental disorders such as ASD or schizophrenia have revealed mutations in other members of the protocadherin family, such as *PCDH15* [42,43,44,45,46]. For example, the *PCDHA* gene cluster, encoding members of the α cluster of the clustered protocadherin subfamily, has been found to be strongly associated with autism. Early data established the role of *PCDHA* in maintaining neuronal survival, synaptic connectivity and axonal convergence [43]. Given the hypothesized connection between autistic features and defects in synaptic plasticity and development, a disruption in the *PCDHA* gene cluster could hence explain its link to autism [43,47]. The non-clustered protocadherins *PCDH9* and *PCDH10*, belonging to the δ1 and δ2 subfamilies, respectively, have also been linked to ASD [44,45,46,48].Marshall et al. (2008) identified gains of copy number variants in intronic regions of *PCDH9* in autistic male and female probands [44]. Inherited copy number variants have also been identified in both *PCDH9* and *PCDH10* in individuals diagnosed with ASD [45]. In addition, impaired social communication and approach have been observed in experimental male mice lacking one copy of *PCDH10* [46]. The structural homology, at the basis of grouping protocadherins, particularly that between *PCDH10* and *PCDH19* that belong to the same δ2 subfamily, further supports a possible and reasonable involvement of the herein-reported *PCDH19* in autism [48]. At the molecular level, the altered function of non-clustered protocadherins would cause neurons to fail to identify other neurons in a circuit because of the absence of protocadherins that act as chemoaffinity labels [49]. *PCDH19* also plays a role in the regulation of intracellular binding proteins, including the GABAA receptor α subunits. As such, it is hypothesized that the absence of *PCDH19* could interfere with the function of other such unidentified binding proteins that are related to autism [33]. Therefore, ASD in males with *PCDH19* mutations could be explained by mechanisms besides the aforementioned cellular interference, which is responsible for EFMR in females and mosaic males [20,33]. 

Interestingly, van Harssel et al. (2013) presented a case of a male with Asperger syndrome who was found to have a missense mutation in *PCDH19* [20]. Unlike the aforementioned case, where the patient was diagnosed at the age of 8.5 years, autistic features in our patients were observed at earlier ages, with the patients’ parents noticing these behaviors at the age of 1.5 years in patient A and 2 years in patient B. Both patient A and the case reported earlier in the literature share the same location of the amino acid change in the 787th residue of protocadherin-19, resulting from the two different DNA mutations in *PCDH19* (c.2360G>T in patient A vs. c.2359C>T in the literature’s case). The normally existing and highly conserved arginine residue present at this location is, however, replaced by a leucine residue in patient A in comparison to cysteine in the literature case [20]. Of note, another hemizygous variant in *PCDH19* was reported in a male with ASD earlier [41]. In that regard, it is noteworthy to mention that *PCDH19* has been recently included in some comprehensive ASD gene panels (https://www.ncbi.nlm.nih.gov/gtr/tests/529181/ accessed on 15 August 2022; Comprehensive Autism Spectrum Disorders Panel (228)-Sema4). Last but not least, while epilepsy in EFMR patients has been shown to follow a peculiar genotype-phenotype expression pattern, it remains unclear whether it also applies to *PCDH19*-linked autism. Reporting further patients with similar presentations is important to confirm the involvement of this gene in ASD in carrier males and to assess whether a genotype-phenotype correlation also exists. Further functional studies are also essential to understand the molecular mechanism of the disease, which may enable, in the future, the implementation of appropriate targeted therapies.

## 5. Conclusions

In conclusion, this is the second report of *PCDH19* variants in males with autism. Reporting further *PCDH19* variants in male patients with autism is important to confirm our hypothesis.

## Data Availability

The datasets used and analyzed during the current study are available from the corresponding author upon a reasonable request.

## Supplementary Materials

The following supporting information can be downloaded at: https://www.mdpi.com/article/10.3390/genes14030598/s1, Table S1: Gene Name.
